# Parameter Estimation and Species Tree Rooting Using ALE and GeneRax

**DOI:** 10.1093/gbe/evad134

**Published:** 2023-07-18

**Authors:** Tom A Williams, Adrián A Davín, Benoit Morel, Lénárd L Szánthó, Anja Spang, Alexandros Stamatakis, Philip Hugenholtz, Gergely J Szöllősi

**Affiliations:** School of Biological Sciences, University of Bristol, 24 Tyndall Ave, Bristol BS8 1TH, United Kingdom; Department of Biological Sciences, Graduate School of Science, University of Tokyo, Tokyo, Japan; Computational Molecular Evolution Group, Heidelberg Institute for Theoretical Studies, Heidelberg, Germany; Institute for Theoretical Informatics, Karlsruhe Institute of Technology, Karlsruhe, Germany; Department of Biological Physics, Eötvös Loránd University, Budapest, Hungary; MTA-ELTE “Lendület” Evolutionary Genomics Research Group, Budapest, Hungary; Institute of Evolution, Centre for Ecological Research, Budapest, Hungary; NIOZ, Department of Marine Microbiology and Biogeochemistry, Royal Netherlands Institute for Sea Research, AB Den Burg, The Netherlands; Computational Molecular Evolution Group, Heidelberg Institute for Theoretical Studies, Heidelberg, Germany; Institute for Theoretical Informatics, Karlsruhe Institute of Technology, Karlsruhe, Germany; Biodiversity Computing Group, Institute of Computer Science, Foundation for Research and Technology Hellas, Heraklion, Greece; The University of Queensland, School of Chemistry and Molecular Biosciences, Australian Centre for Ecogenomics, St Lucia, Queensland, 4072, Australia; Department of Biological Physics, Eötvös Loránd University, Budapest, Hungary; MTA-ELTE “Lendület” Evolutionary Genomics Research Group, Budapest, Hungary; Institute of Evolution, Centre for Ecological Research, Budapest, Hungary

**Keywords:** phylogenetics, gene tree–species tree reconciliation, comparative genomics, microbial evolution

## Abstract

ALE and GeneRax are tools for probabilistic gene tree–species tree reconciliation. Based on a common underlying statistical model of how gene trees evolve along species trees, these methods rely on gene vs. species tree discordance to infer gene duplication, transfer, and loss events, map gene family origins, and root species trees. Published analyses have used these methods to root species trees of Archaea, Bacteria, and several eukaryotic groups, as well as to infer ancestral gene repertoires. However, it was recently suggested that reconciliation-based estimates of duplication and transfer events using the ALE/GeneRax model were unreliable, with potential implications for species tree rooting. Here, we assess these criticisms and find that the methods are accurate when applied to simulated data and in generally good agreement with alternative methodological approaches on empirical data. In particular, ALE recovers variation in gene duplication and transfer frequencies across lineages that is consistent with the known biology of studied clades. In plants and opisthokonts, ALE recovers the consensus species tree root; in Bacteria—where there is less certainty about the root position—ALE agrees with alternative approaches on the most likely root region. Overall, ALE and related approaches are promising tools for studying genome evolution.

**Significance**

Probabilistic gene tree–species tree reconciliation, as implemented in the ALE and GeneRax packages, has emerged as a promising approach to address previously intractable questions in phylogenetics and comparative genomics and has been deployed in several recent studies of early microbial evolution. However, the accuracy of these methods for inferring evolutionary events and rooting species trees was recently questioned. We evaluate these criticisms and find that inferences using ALE and GeneRax are generally accurate in simulations. For empirical datasets, they are in good agreement with alternative approaches and prior biological expectations.

## Introduction

Probabilistic gene tree–species tree reconciliation methods have recently emerged as a powerful approach in phylogenomics and comparative genomics. Recent studies have used reconciliation methods, including the tools ALE (Szöllõsi et al. 2013) and GeneRax (Morel et al. 2020) to infer the root of species trees (Williams et al. 2017; Coleman et al. 2021; Cerón-Romero et al. 2022), map the evolutionary origins of gene families (David and Alm 2011; Martijn et al. 2020; Schön et al. 2022), estimate more accurate single gene trees and ancestral sequence reconstructions (Groussin et al. 2015; Blanquart et al. 2021), and draw conclusions about the contributions of gene gain (Dharamshi et al. 2023), transfer, duplication, and loss to the evolution of bacterial, archaeal (Sheridan et al. 2020, 2022), and eukaryotic (Szöllősi et al. 2015; Harris, Clark, et al. 2022) genomes.

Recently, we applied the ALE reconciliation approach to root the phylogeny of Bacteria (Coleman et al. 2021). By using a model that accounts for transfers, duplications, and losses, we were able to use a substantially greater amount of the available genomic data: 11,272 bacterial gene families, in comparison to the <60 vertically evolving genes that can be used to infer the unrooted tree of life (Harris et al. 2003; Gribaldo et al. 2010; Spang et al. 2015; Hug et al. 2016; Parks et al. 2020; Martinez-Gutierrez and Aylward 2021; Moody et al. 2022) and in the process root the tree of Bacteria. Our analyses support a basal divergence between two major bacterial lineages (clans), the Gracilicutes (Gibbons and Murray 1978) and the Terrabacteria (Battistuzzi et al. 2004; Battistuzzi and Hedges 2009), consistent with other published species trees (Raymann et al. 2015; Taib et al. 2020; Martinez-Gutierrez and Aylward 2021; Aouad et al. 2022; Moody et al. 2022).

However, Tria and Martin (2021) recently suggested that the rates of gene duplication and transfer (and in particular, the ratios of these rates) inferred using ALE are inconsistent with the large excess of gene transfers over duplications in prokaryotic genomes frequently observed in previous analyses (Lerat et al. 2005; Treangen and Rocha 2011; Tria and Martin 2021). In a subsequent paper (Bremer et al. 2022), these and other authors argued that transfer and duplication rate ratios in ALE analyses were unrealistic, and that these biases affect the inference of rooted species trees using the ALE model.

To address these criticisms, we summarize what published analyses using probabilistic reconciliation methods have concluded about variation in the processes of molecular evolution across the tree of life. Overall, these methods recover patterns in good agreement with analyses using other methods, although some limitations are evident. For example, inter-lineage hybridization and incomplete lineage sorting (ILS) are not modeled and likely inflate inferred numbers of gene transfers. We also clarify a number of potential misconceptions about these methods and their results in the recent critiques (Tria and Martin 2021; Bremer et al. 2022), and show that reconciliation-based inferences of the frequencies of duplication, transfer and loss are in good agreement with previous results using other methods. Finally, we re-analyze the bacterial phylogeny using three outgroup-free methods and show that, encouragingly, there is good agreement on the region of the tree most likely to contain the root.

## Results and Discussion

### The Probabilistic Gene Tree–Species Tree Reconciliation Approach Implemented in ALE and GeneRax

Before evaluating the performance of ALE and GeneRax for inferring duplication, transfer and loss events, and rooting species trees, we briefly describe the logic and parameterization of the reconciliation model implemented in these tools, focusing on the elements most relevant to the subsequent analyses. ALE and GeneRax implement a probabilistic model that explains how a gene family can evolve inside a species tree. The process being modeled is one in which a gene family appears on a branch of the species tree and then evolves by means of vertical descent, gene transfer, duplication, and loss. The probabilities of these events are estimated from the data for each gene family.

The key events of the undated ALE/GeneRax DTL model are gene duplication (D), transfer (T), loss (L), and speciation (S), with the root node of the gene tree corresponding to an origination event in the species tree; speciation refers to vertical descent from an ancestral node to its immediate descendant. The probabilities for a gene that is present on a branch of the species tree to experience these events is described by three parameters δ, τ, and λ, each of which can be arbitrarily large positive real numbers. The probabilities of duplication ($P^{D}$), transfer ($P^{T}$), loss ($P^{L}$), or vertical descent (i.e., speciation given by $P^{S}$) on a branch are obtained by dividing the relevant parameters by the sum of the parameters with a default value of 1 corresponding to speciation, that is:


$$P^{D}=\delta /(1+\delta +\tau +\lambda ),$$

$$P^{T}=\tau /(1+\delta +\tau +\lambda ),$$

$$P^{L}=\lambda /(1+\delta +\tau +\lambda )$$

and


$$P^{S}=1-P^{D}-P^{T}-P^{L}.$$

**Fig. 1.**
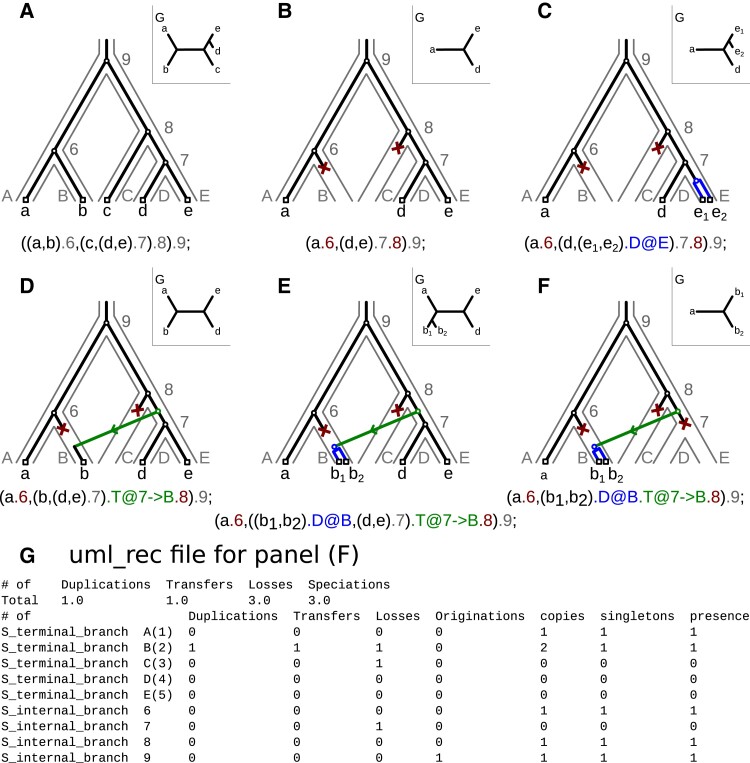
The logic of probabilistic reconciliation, and how to interpret ALE output. Possible reconciliations of different gene trees, given a species tree and the extended Newick string representations for duplication, transfer, loss, and speciation events. The species tree's topology with node names (leaf names and node numbers) is depicted in gray, the gene tree in black (also depicted separately for each case in the top right corner). Evolutionary events needed to reconcile the gene and species trees are highlighted in different colors: red for gene loss, blue for gene duplication, green for gene transfer, and a black circle for speciation. Terminal nodes (leaves or tips) are drawn as black squares. (*A*) The gene tree topology is congruent with the species tree, so no evolutionary events are required to reconcile them. (*B*) The gene tree does not include sequences from species B and C, which can be explained by speciation and loss (SL) events on the species tree. (*C*) Gene duplication (D event) on the branch leading to E. (*D*) Transfer (T event) from branch number 7 to terminal branch B. (*E*) Transfer from branch 7 to branch B and duplication on branch B (DT event). (*F*) All three events at once: a transfer followed by a loss on branch 7 and a duplication on the receiver branch B abbreviated as DTL event. (*G*) The output file *.uml_rec generated by ALEml_undated for the gene tree–species tree reconciliation depicted in (*F*). The uml_rec file contains a summary of the observed evolutionary events, in the case of (*F*) one duplication, one transfer, three losses, and three speciations. After this, a list of Newick strings for each sampled reconciled gene tree follows, in the format shown beneath (*A*)–(*F*). The uml_rec file ends with a description of the frequency of observed events per branch and with other branchwise statistics: branch category, branch name or numeric ID, duplications, transfers, losses, originations, copies, singletons and presence. These events can be summarized (e.g., summed per-branch over all gene families) to compute the total number of events of each type on a branch. We provide scripts to tabulate these summaries in the accompanying Github repository (https://github.com/AADavin/ALEtutorial).

A gene family originates at some internal branch of the species tree, then experiences events according to the above discrete state stochastic process on each subsequent branch, before one or more copies arrive at the tips of the species tree (fig. 1). Note that δ, τ, and λ parametrize the relative probability of vertical descent, D, T, or L on each species tree branch, so they cannot be interpreted as rates (numbers of events that occur per unit time), and are not directly proportional to the number of inferred events. The number and types of events that occur are inferences of the model, not parameters, and are obtained by inspecting the inferred reconciled gene trees (see below). Note also that δ, τ, and λ are estimated separately for each family. The parameters of the model are optimized by jointly maximizing 1) the phylogenetic likelihood on the multiple sequence alignment (MSA) and 2) the likelihood of possible reconciliation scenarios per family. These two components of the joint likelihood—the phylogenetic likelihood and the reconciliation likelihood—can be thought of as expressing two different aspects of the uncertainty of the reconciliation, given an MSA and a species tree. The phylogenetic likelihood captures the relationship between the taxa in the MSA and the gene tree: a given MSA might have evolved on many different gene family trees, each with a different probability. When performing the reconciliation, we need to account for these sources of uncertainty: the uncertainty of the gene tree, given the MSA; and the uncertainty of the reconciliation, given different possible gene trees.

ALE and GeneRax implement different strategies to estimate the reconciled gene trees and the event probabilities. GeneRax searches for the gene tree and the event probabilities that maximize the joint likelihood, computed from the gene sequences and the species tree. ALE approximates the phylogenetic likelihood using conditional clade probabilities computed from a distribution of gene family trees computed in advance. It approximates the joint likelihood by integrating over the conditional clade probabilities, estimates the event probabilities by maximizing this joint likelihood, and samples reconciled gene trees under this joint likelihood.

In both cases, explicit evolutionary scenarios involving a series of gene birth and death events that have given rise to the genes in extant genomes (i.e., *reconciliations*) can be sampled according to their probability. These reconciliations can then be summarized to extract information about the inferred number of gene duplication, transfer, and loss events that occurred during the history of the gene family, and their mapping onto the rooted species tree (see fig. 1).

Simulations, results on real data (Szöllõsi et al. 2013; Scornavacca et al. 2015; Morel et al. 2020), and empirically assayed biochemical properties of ancestrally reconstructed proteins based on alternative gene trees (Groussin et al. 2015) suggest that, by making use of the additional information from the species tree, reconciliation methods including GeneRax and ALE infer more accurate single gene trees than approaches based on the phylogenetic likelihood alone.

The above description explains how parameters are estimated in the context of a fixed, rooted species tree. However, ALE and GeneRax can also be used to root species trees, because different root positions imply different scenarios of gene origination, vertical descent, gain and loss, and therefore different joint (phylogenetic and reconciliation) gene family likelihoods. To test different root positions, the analysis must be run once for each candidate rooted species tree. The gene family likelihoods obtained with each root can then be compared using a tree selection test (such as the Approximately Unbiased [AU] test [Shimodaira 2002]) to identify a confidence set of roots. For example, Coleman et al. (2021) evaluated support for 62 root positions on an inferred unrooted tree of Bacteria, and could reject a root position on all but three adjacent branches (a “root region”) that had the three highest summed gene family log-likelihoods; a step-by-step guide to this procedure was described in a recent book chapter (Harris, Sheridan, et al. 2022); see also the online ALE tutorial at https://github.com/AADavin/ALEtutorial. Of course, the ability of reconciliation likelihoods to choose between candidate roots depends to some extent on the degree to which the model captures the underlying evolutionary dynamics as well as the signal in the data, and we now turn to the critiques of Tria and Martin (2021) and Bremer et al. (2022).

### Do Prior Assumptions Bias the Estimation of Model Parameters in ALE?

**Fig. 2.**
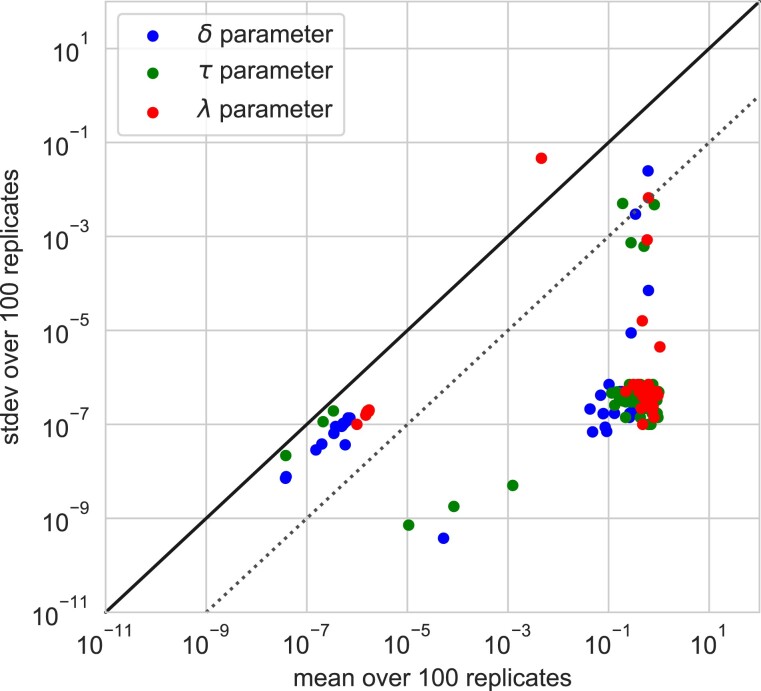
ML estimation of duplication (δ), transfer (τ), and loss (λ) parameters in ALE is robust to the starting values used in the calculation. We sampled 100 gene families randomly from the dataset of Coleman et al. (2021), then estimated δ, τ, and λ parameters 100 times for each family, starting the ML optimization from random initial seeds each time. The plot shows the mean (*x* axis) and SD (*y* axis) of the parameter estimates. The solid line corresponds to SD = mean, while the dashed line denotes SD = 1% of the mean. The results show that δ, τ, and λ parameter estimates are robust to the starting seed values, with SD < mean (typically, SD << mean) in all but a single case (discussed below). The mean SD of the gene family likelihoods across these 100 families was 0.00014 (median 0.0) log-likelihood points. For the single outlier (the loss rate estimated for one family), the mean λ parameter is 0.0046 and the SD is 0.046; for 99/100 replicates, λ ∼ 0 (1 × 10^−10^) with log-likelihood −18.91, whereas in one replicate λ ∼ 0.46 and log-likelihood −18.77, suggesting the optimization algorithm failed to find the ML parameter configuration in this single case.

Tria and Martin (2021) suggested that ALE requires the input of prior δ, τ, and λ rates, while Bremer et al. (2022) claimed that parameter estimates were biased by hard-coded 1:1 τ:δ priors. In fact, model parameters in ALE and GeneRax are estimated via maximum likelihood (ML) optimization without any prior assumptions. For clarity, it is worth noting that some other reconciliation tools do make use of weights for each type of event, which can be set by the user or left as defaults (e.g., the parsimony method RANGER-DTL [Bansal et al. 2018]); however, all of the analyses criticized in Tria and Martin (2021) and Bremer et al. (2022) were performed using ALE, which directly estimates these values from the data. Bremer et al. (2022) further suggested that the default equal initial values for δ and τ—that are required by the Bio++ implementation (Guéguen et al. 2013) of the standard Nelder-Mead optimization algorithm used in ALE for maximizing the likelihood—have an undue influence on the optimized values, although they did not provide any evidence to support the claim. To test whether initial values influence the ML estimates, we sampled 100 gene families at random from the bacterial dataset (Coleman et al. 2021) re-analyzed by Bremer et al., and for each family we estimated the δ, τ, and λ parameters 100 times from different random starting values (chosen independently and uniformly from the interval [0.01,10] for δ, τ, and λ parameters). The results (fig. 2) show that the optimized parameter estimates are highly robust to the starting values, with median standard deviations (SDs) of $8.87\times 10^{-8}$, $3.14\times 10^{-7}$, and $1.00\times 10^{-7}$ in δ, τ, and λ parameters. This indicates that the ML optimization algorithm is able to find the global optimum of the likelihood in terms of the DTL parameters. As in other contexts, best practice may be to repeat the ML procedure several times from random starting values to increase the chance of finding the global optimum, and we note that, for users interested in exploring uncertainty in parameters, an Markov chain Monte Carlo implementation of the ALE algorithm is also available.

### Are ALE-based Estimates of Duplication, Transfer, and Loss Unrealistic?

Some previous studies (Lerat et al. 2005; Treangen and Rocha 2011; Tria and Martin 2021) have suggested that horizontal gene transfer (HGT) is very common in archaeal and bacterial genomes but less frequent in eukaryotes. HGT occurs in eukaryotes (Husnik and McCutcheon 2017; Irwin et al. 2021) but the mechanisms and frequency are debated (Martin 2017; Leger et al. 2018). Rates of HGT also appear to vary across eukaryotic clades: for example, HGT is relatively rare in animals (though interesting cases exist [Kalluraya et al. 2023]), perhaps as a result of the germ–soma distinction (Boto 2014), but appears to be more common in single-celled eukaryotes including Fungi (Richards et al. 2011; Bruto et al. 2014) and Rhizarians (van Hooff and Eme 2023). High-quality genomes from additional eukaryotic groups will likely help to constrain frequencies of HGT more broadly in eukaryotes.

**Fig. 3.**
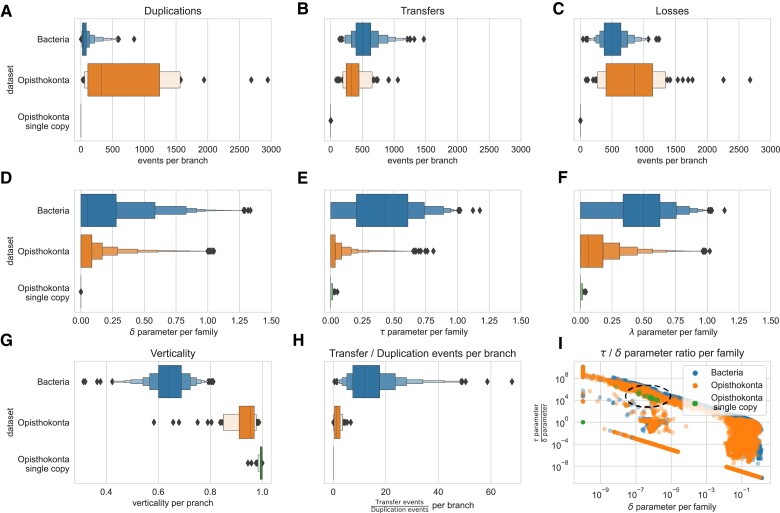
Reconciliation-based estimates of gene transfer, duplication, and loss in the bacterial (Coleman et al. 2021) and opisthokont (Bremer et al. 2022) datasets. ALE reconciliation output files contain a variety of parameter values and inferences, and understanding what each represents is key to interpreting the results. (*A*–*C*) Branchwise estimates of the number of gene duplication, transfer, and loss events in the bacterial and opisthokont datasets. As expected, transfers greatly outnumber duplications in Bacteria, while the numbers of events are more balanced in the opisthokont dataset. Single-copy marker genes in opisthokonts have no inferred duplications, and indeed few transfer, or loss events. (*D*–*F*) δ, τ, and λ parameters for each gene family in the bacterial and opisthokont datasets. While genome dynamics are reflected in the distributions of per-family parameter values (e.g., τ is generally much higher in bacteria than opisthokonts), the between-lineage patterns are less clear because the parameter distributions also reflect an enormous variation in propensity for transfer, duplication, and loss across gene families. Note that parameter values cannot be interpreted as numbers of events, but describe relative probabilities within each gene family. (*G*) Given a species tree and a set of reconciled gene trees, branchwise verticality can be calculated as the number of occurrences of vertical evolution from the ancestral to descendant node, divided by the sum of vertical and horizontal transfer events along the branch (Coleman et al. 2021). Based on ALE estimates, we find that opisthokonts have much higher verticality than Bacteria, as expected (Boto 2014; Ocaña-Pallarès et al. 2022). (*H*) The per-branch ratio of transfer to duplication events inferred by ALE; this is a natural comparator of the per-genome counts of transfer and duplication events reported in previous analyses. As expected, T/D is higher in Bacteria than opisthokonts. Note that T/D is misleading for the opisthokont single-copy orthologous genes because no duplications were inferred in any of the 117 genes in this set. (*I*) The familywise ratio of τ and δ parameter values. This metric is highly variable, both due to biological variation in transfer and duplication frequencies across gene families (Nagies et al. 2020), but also simply because dividing by very low δ parameter values is misleading (note that τ/δ is often very high simply because δ is close to 0; see circled region in [*I*]). Note that (*H*) and (*I*) were conflated in Bremer et al. (2022), leading the authors to conclude that ALE-based ratios of transfer and duplication were unrealistic (see supplementary text, Supplementary Material online for further discussion).

In their critique of ALE, Bremer et al. (2022) suggested that estimated rates of duplication and transfer were biologically unrealistic for two datasets from different domains of life because they failed to capture the expected difference in the relative frequency of transfers and duplications between Bacteria (Coleman et al. 2021) and eukaryotes (Bremer et al. 2022). To investigate, we summarized the ALE output from the two datasets (fig. 3): the bacterial dataset is that of Coleman et al. (2021), the opisthokont dataset is that of Bremer et al. (2022). In the bacterial dataset, the median branchwise number of transfers is an order of magnitude higher than that of duplications (fig. 3), in good agreement with published analyses (Lerat et al. 2005; Treangen and Rocha 2011) and consistent with the expectation that transfers are more frequent than duplications in Bacteria (Tria and Martin 2021). The pattern observed in the opisthokont dataset (Bremer et al. 2022) is quite different. Within Metazoa, ALE infers a large excess of duplications over transfers (median branchwise T/D 0.29), while inferred transfers exceed duplications in Fungi (median branchwise T/D 2.36), though not to the extent observed in the bacterial dataset. These results are consistent with the view that the germ–soma distinction likely acts as a barrier to transfer in animals (Boto 2014), while transfers are more frequent in Fungi (Richards et al. 2011; Ocaña-Pallarès et al. 2022), and even more frequent in Bacteria. Interestingly, a similar contrast in transfer and duplication frequencies between multicellular and unicellular eukaryotes was observed in a recent analysis of 31 streptophyte genomes using ALE (Harris, Clark, et al. 2022): median branchwise T/D was 0.21 for multicellular plants, and 1.11 for their algal relatives (Harris, Clark, et al. 2022).

While the true rate of HGT in Fungi is not known, the absolute number of transfers per branch in Fungi inferred by ALE may nonetheless seem implausibly high (fig. 3). This might result from unmodeled phenomena such as ILS or hybridization, but also from phylogenetic noise, as disagreements between gene and species trees can often most simply be resolved by invoking an HGT event. However, tree topology and root inferences appear robust to ILS in simulations (Morel et al. 2022). More generally, it appears that reconciliation methods tend to infer fewer spurious transfers (and indeed, other kinds of events) than species tree-unaware methods, because reconciled gene trees are more accurate than gene trees inferred from the MSA alone (Szöllõsi et al. 2013; Scornavacca et al. 2015; Morel et al. 2020). For example, when comparing reconciled and species tree-unaware gene trees, we observed 24%, 59%, and 46% reductions in the mean numbers of duplications, transfers, and losses per gene family in an empirical dataset comprising 36 cyanobacterial genomes (Szöllõsi et al. 2013; Morel et al. 2020).

Another factor that is likely to influence the relative number of inferred transfers and duplications is the density of taxon sampling. As pointed out by Tria and Martin (2021), we would expect denser taxon sampling to result in a higher proportion of inferred transfers; this is because, as taxa are sampled more closely, some apparent duplications are revealed to actually be transfers from close relatives. We note that any method that uses phylogenetic trees to distinguish short-distance transfers from duplications (whether by reconciling the gene tree against a species tree, or by parsing gene trees for incongruent relationships) can only succeed when taxon sampling is dense enough to include relatives of the donor, recipient, and enough intermediate taxa to shift the balance of evidence from duplication-followed-by-loss to transfer.

In sum, the conclusion that ALE recovers similar frequencies of transfer and duplication in Bacteria and eukaryotes (Bremer et al. 2022) is incorrect; the model captures a clear difference in transfer frequency between Bacteria and eukaryotes, and between multicellular and unicellular eukaryotes—although the number of inferred transfers is likely somewhat elevated by hybridization and ILS. The suggestion of Bremer et al. (2022) that the frequencies are similar—and another suggestion, that ALE implies an unrealistic increase in genome size over time—may have been due to misinterpretation of the ALE output (conflating model parameters with inferences), as illustrated in figure 3 and discussed in more detail in supplementary text, Supplementary Material online.

### Constraining the Ratio of Duplication and Transfer Parameters to a Predefined Value Reduces Model Fit and Impedes Species Tree Rooting, Particularly for the Most Informative Gene Families

Published analyses using ALE to root species trees have fit the default reconciliation model, in which the probability of duplication, transfer, and loss are modeled using three separate parameters for each gene family, δ, τ, and λ, whose values are inferred using ML. The motivation for this parameterization is that the frequencies of duplication, transfer, and loss vary greatly across gene families (as evident, e.g., in fig. 3).

As part of their critique, Bremer et al. (2022) investigated the impact on species tree root inference of constraining the δ and τ parameters. First, they estimated the parameters of the model using ML, as in the original analyses (Coleman et al. 2021). Using ML, they recovered the same root region as Coleman et al. (2021). On their test dataset of opisthokonts (Fungi and Metazoa), the default approach recovered the expected root (between Fungi and Metazoa) with maximum support. These results support the notion that reconciliation models can recover accurate root information when the model parameters are estimated from the data. For clarity, we note that Bremer et al. (2022) incorrectly refer to ML parameter estimates as “1:1 *T*:*D* ratio” throughout their study; however, the unconstrained δ, τ, and λ parameters are freely estimated via ML and do not, in general, have a 1:1 ratio; see figure 3*I*.

Next, Bremer et al. performed an experiment in which they fixed the ratio of τ and δ parameters. They did not, however, perform ML under a constrained ratio. Instead, they first estimated all three parameters freely by ML, and then performed a second analysis fixing δ as a multiple of the estimated τ (e.g., δ = 0.02 × τ for the 50:1 τ:δ case). The 50:1 ratio was motivated by previous work suggesting that, counted per genome, transfers are about 50 times more frequent than duplications in Bacteria (Tria and Martin 2021). As noted above and in the Supplementary Material online, however, these parameters are estimated familywise and are not interpretable in terms of the number of events. In addition, analysis of this dataset without fixing parameters a priori instead suggests a ∼10-fold excess of transfers over duplications (fig. 3).

For the bacterial dataset, fixing the parameter ratio in this way resulted in a loss of power, with the set of root positions that could not be rejected expanding to include additional nearby branches of the species tree. The same effect was observed on the opisthokont dataset for the 1:2 and 1:50 τ:δ cases. Interestingly, when the rates were fixed to a highly implausible 50-to-1 ratio of τ to δ in opisthokonts, the true root was no longer recovered in the credible set.

In their experiments, Bremer et al. (2022) did not investigate the impact of constraining the δ and τ parameters on model fit. Statistical analysis is usually best done under the best-fitting model (Sokal and Rohlf 2012), and when the model parameters were estimated using ML, ALE recovered the generally accepted fungal–metazoan root in the opisthokont data (Bremer et al. 2022). Therefore, it seems possible that the loss of precision and accuracy observed when constraining the δ and τ parameters might result from poor model fit.

To investigate the impact of fixing parameter ratios on model fit, we first implemented the ability to fix DTL parameter ratios in ALE. Optimizing τ, δ, and λ allows for a valid statistical comparison that is fairer to the simpler model; we hope that this additional function in ALE will also be of use for future investigations of genome evolution. This “fixed TD” model, which fixes the ratio of τ, δ to a user specified value, infers one fewer parameter per gene family than the full model used in the original analyses.

Having implemented the model proposed by Bremer et al., we then compared gene family likelihoods for the 11,272 gene families under the full (independent δ, τ, λ parameters) and restricted model. Model fit was substantially worse under the restricted model; in the opisthokont dataset, fixing δ=2τ resulted in a mean reduction in log-likelihood of 14.6 units per gene family, while in Bacteria fixing $\delta =(1/50{)}_{\tau }$ (the “50:1” ratio) resulted in a mean loss of 20.2 log-likelihood units per family; table 1. Joint estimation of the single δ parameter (with τ set according to the prescribed ratio) and λ by ML using the new implementation in ALE greatly improved the fit of the simple model compared to Bremer et al.'s approximation, although model fit was still significantly worse than the default approach in which δ, τ, and λ are estimated independently (table 1). These results suggest that the loss of power reported by Bremer et al. is due to the use of an overly simple model that fits the real data substantially worse than the default approach.

**Table 1:** Fixing τ:δ Across Gene Families Results in a Significant Loss of Model Fit in the Opsithokont and Bacterial Datasets

τ:δ ratio	Summed LL	Δ LL	Δ LL/family	AIC	Families that reject simpler model (AIC)
Opisthokont dataset (Bremer et al. 2022)					
Free (maximum likelihood DTL estimation)	−507,684.73			**1,062,037**	
Estimate free, then fix δ=2 × $\tau_{\mathrm{opt}}$	−735,742.10	228,057.37	14.66	1,502,596	7,335/15,556
1:2	−534,420.18	26,735.45	1.71	1,099,952	4,817/15,556
1:50	−534,009.37	26,324.64	1.69	1,099,131	4,309/15,556
50:1	−669,876.28	162,191.54	10.4	1,370,865	6,895/15,556
Bacterial dataset (Coleman et al. 2021)					
Free (maximum likelihood DTL estimation)	−2,204,764.16			**4,443,344**	
Estimate free, then fix δ=0.02*$\tau_{\mathrm{opt}}$	−2,432,608.65	227,844.49	20.21	4,887,761	5,930/11,272
50:1	−2,318,604.50	113,840.34	10.1	4,659,753	5,908/11,272
100:1	−2,319,889.26	115,125.09	10.21	4,662,323	5,687/11,272

Bold simply highlights the optimal value (lowest AIC).

For each dataset, the first row shows the summed gene family likelihood when DTL parameters are independently estimated by ML (the default setting, which provides the best overall fit by AIC (bold)). The second row shows the impact of setting δ to twice (opisthokont) or one-fiftieth (bacterial) the value of the value of the τ parameter estimated by ML in the initial analysis (as per Bremer et al. 2022); this results in a large reduction in model fit. Subsequent rows show the log-likelihood summed over families when τ:δ was set to a fixed ratio, but the value of this joint τδ parameter was estimated by ML. AIC was calculated as 2(total number of parameters) − 2(summed log-likelihood); the default approach provides the best model fit (lowest AIC). The final column summarizes the number of families that reject the simpler model on a per-family basis. In the opisthokont dataset, values are computed for 15,556 of the original 15,614 families, because the model could not be fit for 58 families under the 1:50 τ:δ condition due to numerical instability; inferences are very similar for the remaining families under the other three conditions.

To systematically assess model fit on a per-family basis, we used the Akaike Information Criterion (AIC) to compare support for the simpler (two-parameter) vs. the more complex and hence parameter rich (three-parameter) model for each gene family under each condition. This analysis indicated that the AIC rejected the simpler model for 28–52% of gene families across the range of ratios tested (table 1), when considered individually. One contributor to the preference of individual families for the simple or more complex model is family size: in the bacterial dataset, families for which the AIC rejected the simple model tended to be larger (median 11 and mean 47.06 gene copies, compared to median 8 and mean 24.1 for families that did not reject the simple model by AIC; $P=4.84\times 10^{-57}$, Wilcoxon rank-sum test), and family size was strongly correlated with the strength (log-likelihood difference between the simple model and complex model**)** with which the simpler model was rejected (Spearman's ρ=0.23, $P=2.2\times 10^{-16}$). This result suggests that independent estimation of δ, τ, and λ parameters is particularly important for larger gene families, while for the smallest gene families the amount of data does not appear to suffice to reliably optimize them.

To assess whether the larger gene families for which AIC individually rejects the simple (fixed τ:δ ratio) model have distinct rooting information from the smaller families for which the simpler model was not rejected, we divided the families of the bacterial dataset into these two sets and performed an AU root test separately on each. In the original analysis (Coleman et al. 2021), we obtained support for a root region including three branches, corresponding to a root between Gracilicutes and Terrabacteria or on the adjacent branch leading to Fusobacteriota; the analysis did not distinguish whether Fusobacteriota branched as sister to Gracilicutes or to Terrabacteria. The AU test on the 5,930 (fixed two-step procedure) or 5,908 families for which AIC rejected the simple model recovered a root region similar to that inferred from the full dataset, with a root either between Gracilicutes and Terrabacteria or on Fusobacteriota (supplementary fig. S1, Supplementary Material online). Interestingly, this root region contained one fewer branch than the test on the full data, with the branching of Fusobacteriota on the terrabacterial side of the root rejected at *P* < 0.05. That is, the analysis placed additional weight on Fusobacteriota as the earliest-branching group within Gracilicutes, a position that is consistent with analyses of some cell envelope characters (Fusobacteriota possess a Gracilicute-type system for tethering the outer membrane to the cell [Witwinowski et al. 2022]). By contrast, the AU test on the 5,342 smaller gene families for which AIC did not reject the simpler model was much less informative, with a root region including the Gracilicute–Terrabacteria divide (with Fusobacteria branching at the root of Terrabacteria) but also 10 other positions (supplementary fig. S1, Supplementary Material online). In sum, these analyses suggest that the best reconciliation model fit for larger gene families is obtained using the default approach in which δ, τ, and λ are optimized independently, and that such families also contain much of the rooting signal that is available to reconciliation analyses.

The real evolutionary process is more complex than the best available models, and so parameter inferences and analyses under even the full D, T, L model are, to some extent, misspecified. In this context, the experiments of Bremer et al. (2022) on the opisthokont dataset are encouraging. When parameters were estimated from the data, the most plausible root was recovered with maximum support. The main effect of model misspecification appears to be a loss of statistical power, with the model being unable to differentiate between additional branches as fit worsened (table 1). Only when the TD parameters were set to very implausible values (a 50-fold higher τ than δ in animals and Fungi, for all gene families) did the analysis become misleading, in the sense that the expected root was no longer in the 95% credible set. These analyses suggest that the best approach for empirical analyses is to estimate model parameters from the data, rather than setting them to subjective values.

### ALE and Alternative Rooting Methods Capture a Congruent Root Signal for the Bacterial Phylogeny

Given the biological interest of rooting problems, many alternative approaches to outgroup rooting are being developed. One class of methods makes use of branch length information to root trees. Building on the idea of midpoint rooting (rooting a tree in the middle of the longest tip-to-tip path), MAD (Tria et al. 2017) and MinVAR (Mai et al. 2017) are methods that root trees at the position that implies the minimum variation in molecular evolutionary rate from the root to the tips. Molecular clock models (Ho and Duchêne 2014; dos Reis et al. 2016) can also use branch length information to root trees, although in practice these models are not often used for rooting, but rather to infer divergence times on a fixed, rooted species tree. A second class of rooting methods makes use of asymmetric or nonreversible features of the substitution process. For example, the NONREV (Naser-Khdour et al. 2022) and UNREST (Yang 1994) models, implemented in IQ-TREE 2 (Minh et al. 2020) and RootDigger (Bettisworth and Stamatakis 2020), relax the assumption of reversibility in the standard GTR substitution model, so that the instantaneous rate of change from, say, A to G is different to that from G to A. As a result, the likelihood of observing the MSA given the tree also depends on the root of the tree, allowing the root to be inferred without assuming an outgroup. While the best outgroup-free rooting approach is debated and may be dataset-dependent, previous work suggests that all of these approaches can capture root signal and correctly root trees under some conditions (Tria et al. 2017; Bettisworth and Stamatakis 2020; Dombrowski et al. 2020; Wade et al. 2020; Coleman et al. 2021; Naser-Khdour et al. 2022).

**Fig. 4.**
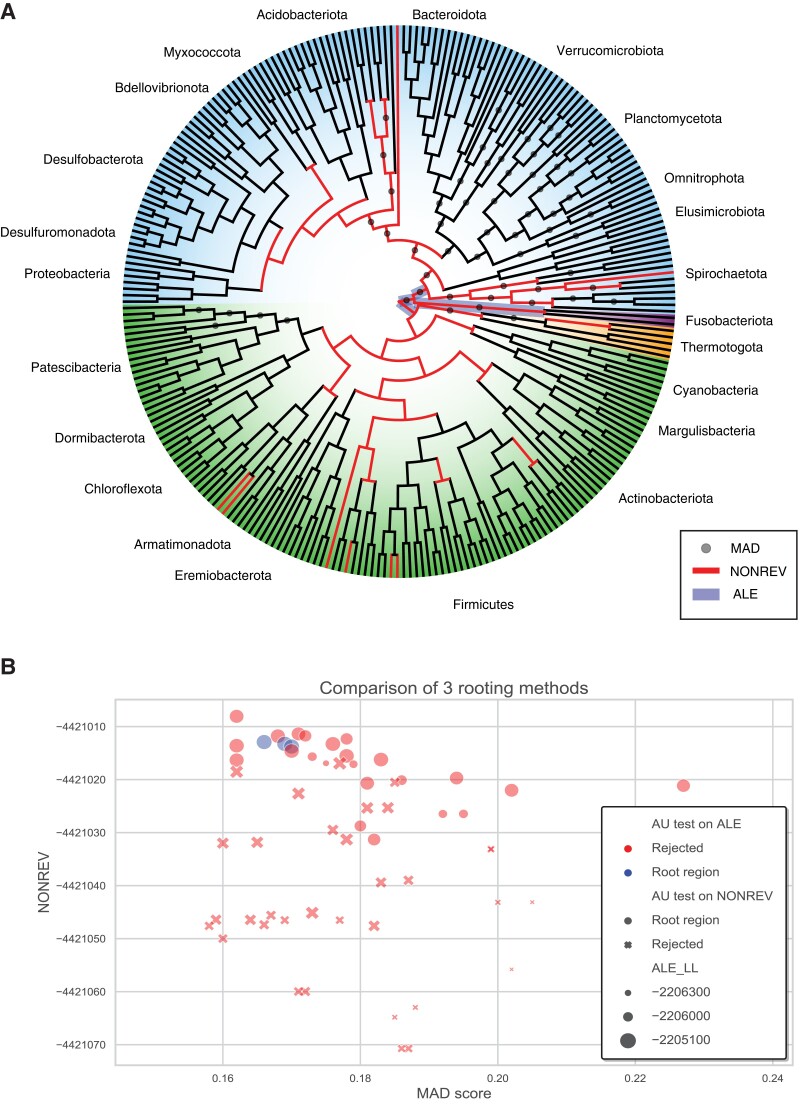
Agreement between reconciliations, branch lengths, and a nonreversible substitution model on the position of the bacterial root. (*A*) An unrooted cladogram of Bacteria indicating root support from ALE, MAD, and NONREV + G. Terrabacteria are highlighted in green, Gracilicutes in blue. For the likelihood-based methods, root positions that could not be rejected by an AU test (*P* < 0.05) are indicated. An AU test using ALE log-likelihoods rejected all but three of the internal branches as a plausible root position, whereas NONREV + G log-likelihoods were more equivocal. This might be because the ALE analysis makes use of more data (11,272 gene families compared to a 62-gene concatenation). For the MAD analysis, we plot the nodes with the 10% lowest (best) AD scores. (*B*) Agreement between MAD scores, ALE reconciliation log-likelihoods, and NONREV + G log-likelihoods for the internal nodes of the bacterial species tree; scores from the three methods are significantly correlated (see main text).

To evaluate the extent of agreement between different outgroup-free rooting approaches on an interesting test dataset, we applied MAD and the nonreversible NONREV + G model to the bacterial dataset we analyzed previously using ALE (Coleman et al. 2021). This dataset is relevant here for two reasons. First, the root of Bacteria is an interesting and debated topic in phylogenetics; second, the critique of Bremer et al. (2022) was in response to our earlier analysis of the bacterial root using ALE. As shown in figure 4, root support is significantly correlated between ALE, MAD, and NONREV + G, with all three approaches favoring a similar set of root positions. We compared the values from the three methods using the Spearman's rank correlation, finding a ρ value different from zero in all three cases (MAD vs. NONREV: ρ=−0.27, *P* = 0.03; MAD vs. ALE: ρ=−0.48, $P=5.56\times 10^{-5}$; NONREV vs. ALE: ρ=0.71, $P=1.03\times 10^{-10}$; note that for MAD scores, smaller is better, while for likelihoods larger is better). Of the two probabilistic methods, ALE has greater power to reject root positions with lower log-likelihoods using an AU test (Shimodaira 2002; fig. 4). This might reflect the difference in the nature of root signal being captured by these two approaches: the summed ALE log-likelihood pools root signal from reconciliations across a large number of gene families (11,272 gene families in this case), while the NONREV + G log-likelihoods summarize the information about the nonreversibility of the substitution process in the 62-gene concatenated alignment. However, given that this is an empirical dataset—the root of Bacteria is not known with certainty—it is difficult to exclude the possibility that the result might also reflect over-confidence of the reconciliation model in choosing among statistically similar alternatives. It is therefore encouraging that analyses of simulated data, and of empirical datasets where there is greater biological consensus on the true root position, also suggest that rooting based on reconciliation likelihoods is accurate. In particular, Williams et al. (2017) analyzed gene families simulated under a more complex DTL model and found that the ALE ML root was the true root in 19/20 replicates; in the final replicate, the ML root was one branch away from the true root. Recent empirical analyses of land plants (Harris, Clark, et al. 2022) and opisthokonts (Bremer et al. 2022) using ALE recovered plausible root positions that have been recovered in many other analyses—between bryophytes and tracheophytes for plants (One Thousand Plant Transcriptomes Initiative 2019), and between animals and Fungi for opisthokonts (Torruella et al. 2012).

As the reconciliation root support is summed over many gene families, it is possible to dissect the nature of the root signal by evaluating how families with different properties differ in root support. This may be a useful robustness check in rooting analysis, similar to filtering out fast-evolving, poorly aligned or compositionally biased sites from an alignment in traditional phylogenetics. In Coleman et al. (2021), we ranked gene families by a range of different metrics, then sequentially removing families and evaluating the impact on root support. These analyses indicate that broadly distributed gene families (i.e., families found in many taxa) and predominantly, but not entirely, vertically evolving gene families were the most informative, because removing these families from the dataset reduced the difference between the log-likelihood scores of the different root positions and hence the discriminatory power. For example, filtering out the top 20% of mcl gene families ranked by verticality or breadth of distribution in extant Bacteria (number of genomes encoding the gene family) greatly reduced the likelihood difference among candidate root positions, while removing the bottom 20% of gene families by these criteria had a negligible effect; see Figure S12 in Coleman et al. (2021). Since such families are expected to have originated early along the species tree, this finding suggests that the root signal is driven by D, T, and L events on the deeper branches of the species tree.

## Conclusion

In this study, we evaluated several recent critiques of the probabilistic gene tree–species tree reconciliation model implemented in ALE and GeneRax, and conclude that they are unfounded. ML parameter estimates are not affected by starting seed values and, as in any ML analysis, do not make use of priors. ALE recovers the major differences in numbers of transfers and duplications expected when comparing prokaryotes and eukaryotes, and when comparing unicellular and multicellular eukaryotes—although the number of inferred transfers is likely to be inflated by nonmodeled processes such as ILS, and the numbers of events are likely influenced by the density of taxon sampling. When the reconciliation model is fit by ML, it recovers the true root in simulations and the expected root in empirical cases such as the phylogeny of animals and Fungi, where there is biological consensus on the root position. For the species tree of Bacteria—an interesting dataset where the root is not known with certainty—there is encouraging agreement between ALE and other outgroup-free rooting methods including MAD and NONREV + G. Our analyses caution against arbitrarily fixing parameters or their ratios, which leads to worse model fit and performance (table 1) in comparison to the default ML procedure.

While already useful and complementary to traditional phylogenetic analyses, the reconciliation model implemented in ALE and GeneRax is certainly not consummate. In addition to the limitations discussed above, an important simplifying assumption is that the same δ, τ, and λ parameters apply to all branches of the species tree. This assumption is certainly violated by real data: for example, vertically inherited endosymbionts and intracellular parasites often undergo extensive gene loss compared to their free-living relatives (McCutcheon and Moran 2011), while multicellular eukaryotes are commonly assumed to acquire fewer genes by horizontal transfer than do their unicellular relatives. Since inferred transfers, duplications, and losses ultimately depend on the gene tree topologies, reconciliation analyses can recover these broad patterns in the variation of D, T, and L across clades (e.g., the higher T/D in Bacteria than Opisthokonts, and in Fungi compared to Metazoa—fig. 2, this study; but also [Ocaña-Pallarès et al. 2022]). However, the assumption of a constant branchwise probability means that the method lacks the power to identify precisely where major shifts in the frequency of duplications, transfers, or losses occur. In ALE, it is currently possible to test hypotheses about branchwise shifts in D, T, or L parameters by applying multipliers to specific branches of interest, and current work is focused on implementing “highways” of transfer between distant points on the species tree. However, a more general solution, involving optimization of parameters across branches and gene families, remains intractable. A first step in this direction would be to introduce a mixture model with a few branch specific categories.

Overall, our results suggest that each of the outgroup-free rooting methods considered above (rooting using reconciliations, branch lengths, and a nonreversible substitution model) is capturing different aspects of a genuine root signal. The degree of agreement observed is particularly encouraging given that the three methods make use of largely distinct sources of root information, and suggests that analyses combining different types of root information are a promising direction for future progress. For example, ALE could be used to reconcile gene tree distributions rooted using MAD, NONREV, or UNREST. New approaches combining existing methods, and addressing some of the limitations of the current implementations, might be useful for making progress on cases where there is less community consensus on the root of the tree. For some of these interesting problems, there has recently been some encouraging agreement between reconciliation and more traditional phylogenetic approaches, but there is still enormous potential for progress. For example, in the context of bacterial phylogeny, the placement of the genome-reduced Patescibacteria (Candidate Phyla Radiation [Brown et al. 2015]) as sister to the Chloroflexota + Dormibacterota within the Terrabacteria has recently gained support from both standard phylogenetic (Taib et al. 2020; Martinez-Gutierrez and Aylward 2021; Moody et al. 2022) and reconciliation-based (Coleman et al. 2021) approaches. A bacterial root at, or near, a deep divide between Gracilicutes and Terrabacteria has also received support from both reconciliation and outgroup-rooted analyses (Battistuzzi and Hedges 2009; Raymann et al. 2015; Coleman et al. 2021; Martinez-Gutierrez and Aylward 2021; Aouad et al. 2022; Moody et al. 2022). A putative archaeal root between at least some DPANN clades and other Archaea has been recovered both in reconciliation (Williams et al. 2017) and more traditional analyses (Dombrowski et al. 2020; Martinez-Gutierrez and Aylward 2021; Aouad et al. 2022). As phylogenetic methods improve and new lineages of Archaea and Bacteria are discovered, the roots of major microbial radiations will continue to be tested.

## Methods

### Data Provenance

For re-analyses of the Coleman et al. (2021) dataset, we downloaded the .ale files for mcl gene families, the candidate rooted species trees, and the 62-gene amino acid concatenate from the original data repository (https://doi.org/10.6084/m9.figshare.12651074.v12). The .ale format concisely summarizes information on the bipartitions from a sample of bootstrap or mcmc trees. The analyses underpinning figure 3 were performed on SpeciesTree_528, although results were similar for the other two branches of the root region. The rooted species tree and .ale files for the 31-genome streptophyte dataset were obtained from the original repository (https://doi.org/10.6084/m9.figshare.c.5682706.v1). The .ale files for the opisthokont dataset analyzed by Bremer et al. (2022) were provided by the authors of that study.

### Root Inference Using ALE, MAD, and NONREV + G

To facilitate direct comparison between methods, all root analyses were performed on the unrooted species tree topology inferred from the 62-gene concatenate in Coleman et al. (2021) under the best-fitting LG + C60 + R8 + F model using IQ-TREE 1.6.10 (Nguyen et al. 2015). ALE log-likelihoods for each root position on the bacterial species tree were obtained from the original analyses. The NONREV + G model was fit to the alignment in a single partition using IQ-TREE 2 (Minh et al. 2020; Naser-Khdour et al. 2022). We used the –root-test option to evaluate the likelihood of each possible root position. The AD values for each branch were estimated using the Python implementation of the MAD algorithm (Tria et al. 2017).

### Updates to ALE Code

The new option to fix δ, τ, and λ parameters to a given ratio was implemented in ALE (https://github.com/ssolo/ALE) and is available in the current release. Any ratio of two parameters can be fixed by setting, for example, DT = 0.02 (to fix τ to be 50 times δ).

### ALE Analyses With Fixed Parameters

To fit the ALE model with fixed parameters, we ran ALEml_undated fixing the ratios as indicated in table 1. The .uml_rec output files are provided in the data repository for this manuscript (10.5281/zenodo.7682207).

## Supplementary Material

evad134_Supplementary_Data

## Data Availability

Supplementary data for this manuscript are available in the Zenodo.org repository at DOI: 10.5281/zenodo.7682207.
